# RhoU forms homo-oligomers to regulate cellular responses

**DOI:** 10.1242/jcs.261645

**Published:** 2024-01-30

**Authors:** Natasha S. Clayton, Richard G. Hodge, Elvira Infante, Dominic Alibhai, Felix Zhou, Anne J. Ridley

**Affiliations:** ^1^ School of Cellular and Molecular Medicine, University of Bristol, Biomedical Sciences Building, University Walk, Bristol BS8 1TD, UK; ^2^ Randall Centre for Cell and Molecular Biophysics, King's College London, Guy's Campus, London SE1 1UL, UK; ^3^ Wolfson Bioimaging Facility, University of Bristol, Biomedical Sciences Building, University Walk, Bristol BS8 1TD, UK; ^4^ Ludwig Institute for Cancer Research, Nuffield Department of Clinical Medicine, University of Oxford, Oxford OX3 7DQ, UK; ^5^ Lyda Hill Department of Bioinformatics, University of Texas Southwestern Medical Center, Dallas, TX 75390, USA

**Keywords:** RhoU, Rho GTPases, Cell morphology, Phosphorylation, p21-activated kinase, PAKs

## Abstract

RhoU is an atypical member of the Rho family of small G-proteins, which has N- and C-terminal extensions compared to the classic Rho GTPases RhoA, Rac1 and Cdc42, and associates with membranes through C-terminal palmitoylation rather than prenylation. RhoU mRNA expression is upregulated in prostate cancer and is considered a marker for disease progression. Here, we show that RhoU overexpression in prostate cancer cells increases cell migration and invasion. To identify RhoU targets that contribute to its function, we found that RhoU homodimerizes in cells. We map the region involved in this interaction to the C-terminal extension and show that C-terminal palmitoylation is required for self-association. Expression of the isolated C-terminal extension reduces RhoU-induced activation of p21-activated kinases (PAKs), which are known downstream targets for RhoU, and induces cell morphological changes consistent with inhibiting RhoU function. Our results show for the first time that the activity of a Rho family member is stimulated by self-association, and this is important for its activity.

## Teaser

**Highlighted Article:** The atypical Rho GTPase RhoU self-associates via its C-terminal extension. This interaction increases activation of downstream p21-activated kinases and regulates cell morphology, constituting a new mechanism of Rho GTPase regulation.

## INTRODUCTION

The Rho family of small guanosine triphosphates (GTPases) form a subfamily of the Ras superfamily of small GTPases. Rho GTPases regulate multiple cellular processes through their effects on cytoskeletal and cell adhesion dynamics, including cell migration, cytokinesis, cell cycle progression, vesicle trafficking and transcriptional changes (Hodge and Ridley, 2016; Ridley, 2015). Most Rho GTPases cycle between an active GTP-bound conformation and an inactive GDP-bound conformation. When bound to GTP, they interact with effector proteins to induce cellular responses. The intrinsic ability of Rho GTPases to exchange GDP for GTP is catalysed by guanine nucleotide exchange factors (GEFs), leading to their activation (Rossman et al., 2005). Conversely, GTPase-activating proteins (GAPs) stimulate GTP hydrolysis, inactivating them (Hodge and Ridley, 2016). Rho GTPases are also regulated by multiple different types of post-translational modifications, including prenyl and palmitoyl lipidation, phosphorylation and ubiquitylation (Hodge and Ridley, 2016). Some Rho GTPases are also regulated by guanine nucleotide dissociation inhibitors (GDIs), which interact with their C-terminal prenyl groups, extract them from membranes and sequester them in the cytosol (Garcia-Mata et al., 2011).

RhoU is an atypical Rho GTPase that is part of the RhoU and RhoV subfamily of Rho GTPases (Hodge and Ridley, 2017). It has unique N- and C-terminal extensions not found in other Rho GTPases, and the N-terminal extension region binds to SH3 domains from Grb2, Nck and phospholipase Cγ (Shutes et al., 2004). Grb2 binding increases its activity in cells (Shutes et al., 2004). The C-terminal extension region is modified by Src-mediated tyrosine phosphorylation, which decreases its plasma membrane association and activity (Alan et al., 2010). RhoU is C-terminally palmitoylated and not prenylated, and hence will not bind to RhoGDIs (Berzat et al., 2005). In addition, RhoU has a 10-fold higher intrinsic guanine nucleotide exchange rate *in vitro* than Cdc42, whereas the intrinsic GTP hydrolysis rate of RhoU and Cdc42 is similar (Shutes et al., 2004). Like Cdc42, it interacts with p21-activated kinases (PAKs) and PAR6, but does not bind to the Cdc42 target N-WASP (Brady et al., 2009; Tao et al., 2001). Although no GEFs or GAPs for RhoU have so far been identified, it is probably regulated by GAPs because a constitutively active mutant, RhoU-Q107L, is more active than wild-type RhoU in binding and activating PAK1 and stimulating cellular responses (Berzat et al., 2005; Saras et al., 2004; Shutes et al., 2004). RhoU is also regulated at the transcriptional level, it was first identified as a Wnt-inducible gene (Tao et al., 2001), and its expression is induced by Notch1 and STAT3 (Bhavsar et al., 2013; Schiavone et al., 2009). Interestingly, PAK4 protects RhoU from ubiquitin-mediated degradation through a mechanism not requiring PAK4 kinase activity (Dart et al., 2015).

*RhoU* expression is upregulated in prostate cancer and has been suggested to be a prognostic indicator for prostate cancer progression, along with other genes (Alinezhad et al., 2016; Corradi et al., 2021; De Piano et al., 2020). Knockdown of RhoU expression by RNAi in prostate cancer cell lines reduces migration and invasion (Alinezhad et al., 2016; Tajadura-Ortega et al., 2018). RhoU is also implicated in cell adhesion. For example, RhoU localizes to focal adhesions in HeLa cells and fibroblasts and stimulates loss of focal adhesions in both cell types (Chuang et al., 2007; Ory et al., 2007). Consistent with these results, RhoU depletion in breast and prostate cancer cell lines increases focal adhesion size (Dart et al., 2015; De Piano et al., 2020).

Here, we investigate the hypothesis that RhoU is regulated by novel mechanisms given that it is an atypical GTPase. We find that RhoU homodimerizes in prostate cancer cells and that this is mediated by its C-terminal extension. This interaction is important for activation of PAKs, which are kinases activated by RhoU as well as other Rho family GTPases. Our results show that RhoU activity is stimulated by self-association.

## RESULTS AND DISCUSSION

### RhoU overexpression promotes PC3 prostate cancer cell migration and invasion

**Fig. 1.**
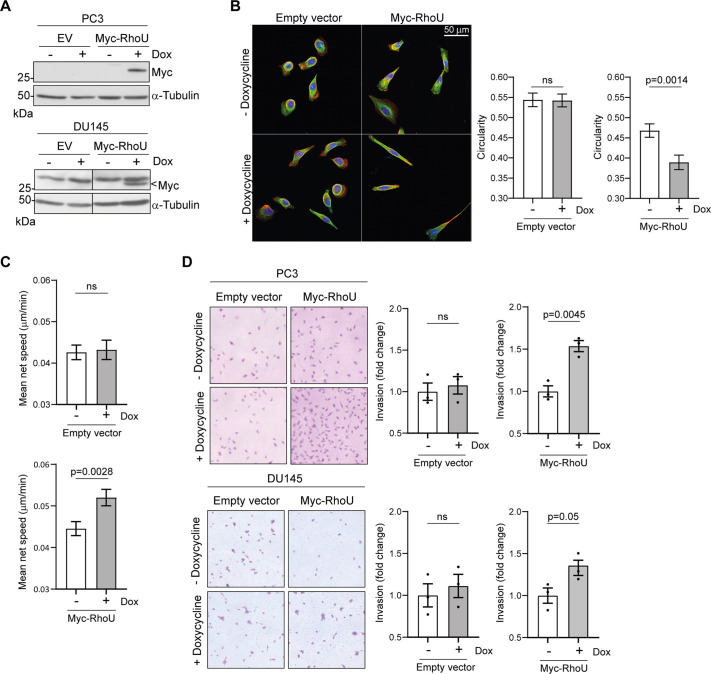
**Overexpression of RhoU increases PC3 cell migration and invasion.** (A) PC3 and DU145 cells harboring a doxycycline-inducible Myc-RhoU construct or empty vector (EV) were incubated for 16 h with or without 1 mg/ml doxycycline (Dox) before lysates were immunoblotted with an anti-Myc antibody. α-Tubulin was used as a loading control. Data are representative of three independent experiments. (B) PC3 doxycycline-inducible cell lines were seeded onto fibronectin-coated glass coverslips and incubated for 24 h with or without 1 mg/ml doxycycline prior to fixation and staining for F-actin (Alexa Fluor 546–phalloidin; red), tubulin (FITC-labelled anti-tubulin antibody; green) and DNA (Hoechst 33342; blue). Images are representative of three independent experiments. Scale bar: 50 µm. Circularity was calculated for 120 cells per condition, across three experiments. Data are presented as mean±s.e.m. *P*-values were determined by Mann–Whitney test. (C) PC3 doxycycline-inducible cell lines were incubated for 16 h with or without 1 mg/ml doxycycline prior to time-lapse microscopy. Cells were imaged every 6 min for 24 h using a 20× objective and migration tracks were generated using the MOSES framework. Data are presented as mean net migration speed of all cells within each field of view. Error bars represent s.e.m. *P*-values were determined by Mann–Whitney test. Data are representative of three independent experiments. (D) PC3 and DU145 doxycycline-inducible cell lines were seeded onto Matrigel-coated Transwell inserts in serum-free medium with or without 1 mg/ml doxycycline. Serum-free medium supplemented with 10 ng/ml HGF with or without 1 mg/ml doxycycline was added to the basal chamber. After 24 h, cells adhered to the underside of the filter were stained with Crystal Violet. Images are representative of three independent experiments. Data are presented as mean±s.e.m. *P*-values were determined by an unpaired two-tailed *t*-test. ns, not significant.

RhoU overexpression has been reported to promote the formation of filopodia and decrease focal adhesions in fibroblasts (Ory et al., 2007; Saras et al., 2004), but the effect of RhoU overexpression in prostate cancer cells has not been tested. Overexpression of Myc–RhoU using a doxycycline-inducible expression construct (Fig. 1A) caused PC3 prostate cancer cells to adopt an elongated phenotype (Fig. 1B, reduced circularity in graph) and resulted in an increased speed of 2D cell migration (Fig. 1C). Myc–RhoU overexpression also increased the invasion of PC3 cells through Matrigel, an effect which was recapitulated in a second prostate cancer cell line, DU145 (Fig. 1D). Given that RhoU depletion increases the size of paxillin-positive adhesions in PC3 cells (De Piano et al., 2020), we reason that RhoU overexpression promotes PC3 cell migration and invasion through increased focal adhesion turnover.

### RhoU forms homomeric complexes at membranes

**Fig. 2.**
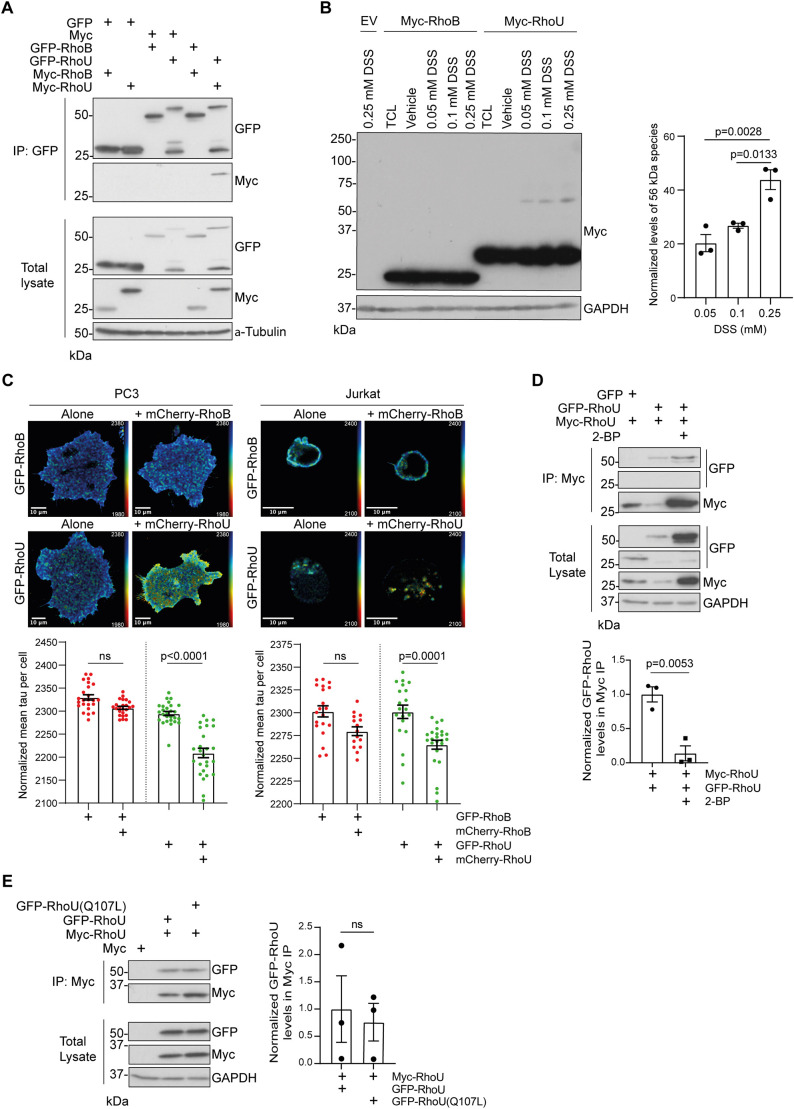
**RhoU forms homo-oligomeric complexes at membranes.** (A) Lysates from COS7 cells transfected with pEGFP-C1, pEGFP-C1-RhoU/RhoB, pRK5-myc and pRK5-myc-RhoU/RhoB were immunoprecipitated (IP) with GFP-Trap^®^ agarose beads. Samples were immunoblotted using anti-GFP and anti-Myc antibodies. α-Tubulin was used as a loading control. Data are representative of three independent experiments. (B) Lysates from COS7 cells transfected with pRK5-myc empty vector (EV), pRK5-myc-RhoB or pRK5-myc-RhoU were either left untreated (TCL; total cell lysate) or incubated with vehicle control or DSS. Lysates were immunoblotted using an anti-Myc antibody. GAPDH was used as a loading control. Graph shows mean±s.e.m. values from three independent experiments. Levels of the ∼56 kDa species were normalized against GAPDH levels. *P*-values were determined by one-way ANOVA with Tukey's multiple comparisons post test. (C) Left, PC3 cells transfected with pEGFP-C1-RhoU/RhoB and pmCherry-C1-RhoU/RhoB were seeded on fibronectin-coated glass and fixed after 4 h. Right, Jurkat cells nucleofected with pEGFP-C1-RhoU/RhoB and pmCherry-C1-RhoU/RhoB were seeded on fibronectin-coated glass in the presence of 1 ng/ml CXCL12 and fixed after 15 min. Fluorescence lifetime of GFP is depicted using a pseudocolor scale (blue, normal lifetime; red, FRET). FLIM images are representative of three independent experiments. Graphs show mean±s.e.m. tau (lifetime) values per cell and individual FRET values for at least 15 cells per condition, from three independent experiments. *P*-values were determined using one-way ANOVA with Sidak's multiple comparisons post test. (D) COS7 cells transfected with pRK5-myc-RhoU and either pEGFP-C1 or pEGFP-C1-RhoU were treated with DMSO or 100 µM 2-BP for 2 h. Lysates were immunoprecipitated using Myc-TRAP^®^ agarose beads and samples were immunoblotted using anti-GFP and anti-Myc antibodies. GAPDH was used as a loading control. Graph shows mean±s.e.m. normalized levels of GFP–RhoU in Myc immunoprecipitates from three independent experiments GFP–RhoU levels in IP samples were normalized against total GFP–RhoU and immunoprecipitated Myc–RhoU levels. *P*-values were determined by an unpaired two-tailed *t*-test. (E) Lysates from COS7 cells transfected with pRK5-myc, pRK5-myc-RhoU, pEGFP-C1-RhoU and pEGFP-C1-RhoU(Q107L) were immunoprecipitated with Myc-Trap^®^ agarose beads. Samples were immunoblotted using anti-GFP and anti-Myc antibodies. GAPDH was used as a loading control. Data are representative of three independent experiments. Graph shows mean±s.e.m. normalized levels of GFP–RhoU in Myc immunoprecipitates from three independent experiments. GFP–RhoU levels in IP samples were normalized against total GFP–RhoU levels and immunoprecipitated Myc–RhoU levels. *P*-values were determined by an unpaired two-tailed *t*-test. ns, not significant.

In experiments aimed at identifying novel RhoU targets, we discovered that RhoU associated with itself by performing co-immunoprecipitation with differently tagged RhoU proteins (Fig. 2A). No self-association was detected for another Rho family member, RhoB, which was chosen as a control because, like RhoU, it is known to localize to endosomes and the plasma membrane and to be palmitoylated as well as isoprenylated (Adamson et al., 1992; Phuyal and Farhan, 2019). RhoU oligomerization was further demonstrated using a cross-linking approach in Myc–RhoU-expressing COS7 cells (Fig. 2B). Disuccinimidyl suberate (DSS) is a membrane-permeable cross-linker that contains two amine-reactive N-hydroxysuccinimide ester groups either side of an eight-carbon spacer arm. On the addition of increasing concentrations of DSS, a dose-dependent increase in a higher molecular mass cross-linked Myc–RhoU species was observed (Fig. 2B). This cross-linked Myc-RhoU species migrated at ∼56 kDa on SDS-PAGE gels, which is equivalent to two 28-kDa RhoU monomers. This suggested RhoU can dimerize, which was unexpected as classical Rho GTPases are assumed to be monomeric based on crystal structures (Ihara et al., 1998; Mott and Owen, 2018; Worthylake et al., 2000). By contrast, no dimers of Myc–RhoB were detected following DSS treatment (Fig. 2B).

To investigate whether RhoU self-associates in living cells, PC3 cells were co-transfected with GFP–RhoU and mCherry–RhoU or GFP–RhoB and mCherry–RhoB as controls. Cells were analysed by fluorescence-lifetime imaging microscopy (FLIM) to determine whether the differently fluorescent-protein-tagged proteins were close enough for FRET to occur (<10 nm; Day and Davidson, 2012). GFP–RhoU and mCherry–RhoU demonstrated FRET, but not GFP–RhoB and mCherry–RhoB (Fig. 2C). These results indicate that self-association is specific to RhoU and not a consequence of high Rho GTPase expression. We have previously shown that RhoU expression is upregulated in T-cell acute lymphoblastic leukemia (T-ALL) cells (Bhavsar et al., 2013). Using FLIM, we found that RhoU self-association could also be detected in Jurkat cells, a T-ALL cell line (Fig. 2C). In PC3 cells, RhoU interacted with itself primarily at the plasma membrane, whereas in Jurkat cells, RhoU oligomers localized to intracellular sites (Fig. 2C). Previous work has demonstrated that the palmitate analog 2-bromopalmitate (2-BP) causes mislocalization and cytosolic accumulation of RhoU (Berzat et al., 2005). Addition of 2-BP to cells resulted in a significant decrease in the co-immunoprecipitation of differently tagged RhoU proteins (Fig. 2D). This indicates that the dimerization of RhoU is dependent on its ability to target to membranes. Interestingly, 2-BP treatment increased levels of GFP- and Myc-tagged RhoU, suggesting that RhoU might be stabilized by mislocalization to the cytoplasm (Fig. 2D).

To test whether RhoU dimerization requires RhoU to be in its active GTP-bound form, we used a RhoU-Q107L mutant, analogous to the activating Q61L mutation in Ras, which locks Ras in the active GTP-bound conformation (Ory et al., 2007). The Q107L mutation had no effect on the ability of RhoU to dimerize with wild-type RhoU (Fig. 2E), indicating that the GTP-binding domain does not play a crucial role in RhoU self-association.

**Fig. 3.**
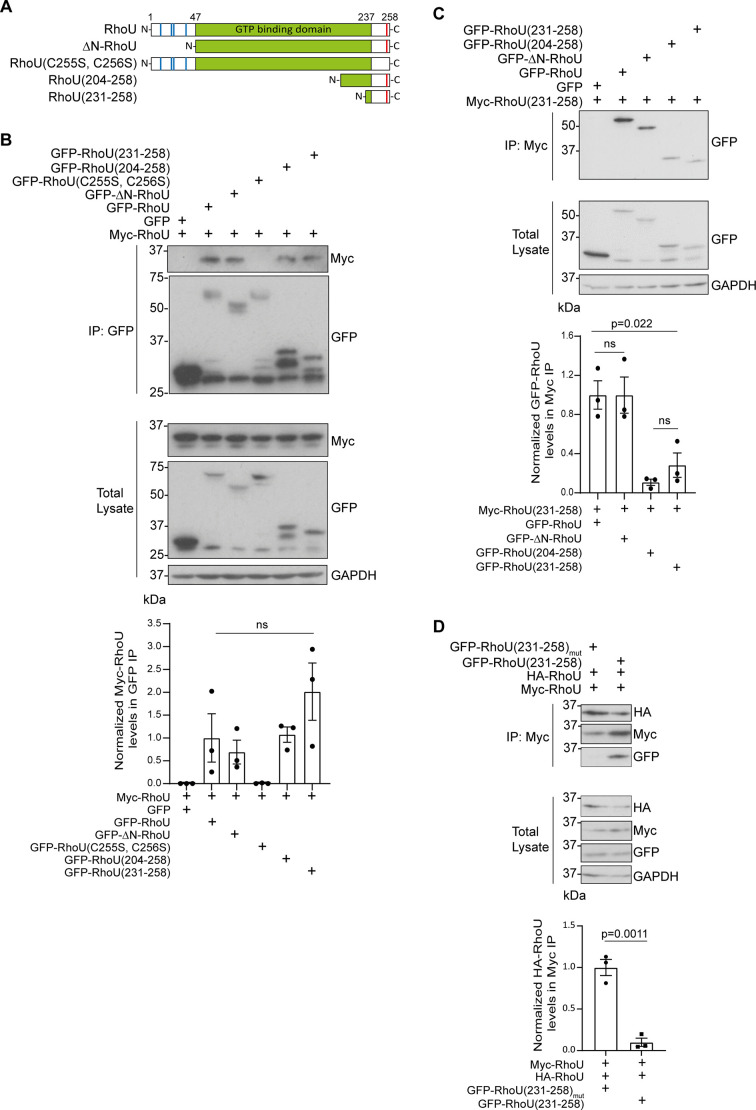
**RhoU homo-oligomerization is mediated by the C-terminal extension.** (A) Schematic representation of RhoU mutants ΔN-RhoU(48–258), RhoU(C255S, C256S), RhoU(204–258) and RhoU(231–258). Blue lines indicate proline-rich motifs; red lines indicate S-palmitoylation sites. (B) Lysates of COS7 cells co-expressing GFP or GFP–RhoU truncation mutants with Myc–RhoU were immunoprecipitated using GFP-TRAP^®^ agarose beads and samples were immunoblotted using anti-GFP and anti-Myc antibodies. GAPDH was used as a loading control. Graph shows mean±s.e.m. values from three independent experiments. Myc–RhoU levels in IP samples were normalized against total Myc–RhoU levels and immunoprecipitated GFP–RhoU levels. *P*-values were determined by one-way ANOVA with Tukey's multiple comparisons post test. (C) COS7 cells were transfected with pRK5-myc-RhoU(231-258) and pEGFP-C1 empty vector, pEGFP-C1-RhoU, pEGFP-C1-ΔN-RhoU (48-258), pEGFP-C1-RhoU(204-258) or pEGFP-C1-RhoU(231-258). Lysates were immunoprecipitated using Myc-TRAP^®^ agarose beads and samples were immunoblotted using anti-GFP and anti-Myc antibodies. GAPDH was used as a loading control. Graph shows mean±s.e.m. values from three independent experiments. GFP–RhoU levels in IP samples were normalized against total GFP–RhoU levels and immunoprecipitated Myc–RhoU(231–258) levels. *P*-values were determined by one-way ANOVA with Tukey's multiple comparisons post test. (D) Lysates of COS7 cells expressing Myc–RhoU and HA–RhoU with either GFP–RhoU(231–258) or GFP–RhoU(231–258)_mut_ were immunoprecipitated using Myc-TRAP^®^ agarose beads and samples were immunoblotted using anti-GFP and anti-Myc antibodies. GAPDH was used as a loading control. Graph shows mean±s.e.m. values from three independent experiments. HA–RhoU levels in IP samples were normalized against total HA–RhoU levels and immunoprecipitated Myc–RhoU levels. *P*-values were determined by an unpaired two-tailed *t*-test. ns, not significant.

RhoU has unique N-terminal and C-terminal extensions, which are not related to similar extensions in other members of the Rho GTPase family such as RhoV and Rnd proteins (Fig. 3A) (Hodge and Ridley, 2017). We considered that the N- and C-terminal regions were likely mediators of dimerization. The C-terminal four amino acids of RhoU are CCFV; S-palmitoylation of RhoU at the second C-terminal C256 has been shown to be crucial for its association with biological membranes, although both C255 and C256 contribute to RhoU subcellular localization (Berzat et al., 2005). We found that RhoU(C255S, C256S) localized to the cytoplasm (data not shown) and failed to co-immunoprecipitate with wild-type RhoU (Fig. 3B). We reasoned that deletion of the RhoU C-terminal extension would result in a similar mislocalization of RhoU and therefore generated two constructs expressing different lengths of the C-terminal extension in isolation (Fig. 3A). Both RhoU(204–258) and RhoU(231–258) co-immunoprecipitated with full-length RhoU to the same extent as the wild-type protein (Fig. 3B), indicating that RhoU dimerization is mediated by residues between amino acids 231 and 258. We found that deletion of the N-terminal extension had no effect on RhoU dimerization (Fig. 3B).

Further co-immunoprecipitation experiments revealed that RhoU(231–358) was able to self-associate, although this interaction was significantly weaker than its interaction with full-length RhoU (Fig. 3C). We therefore suggest that RhoU dimerization is mediated by self-association of the RhoU C-terminal extension but requires a region within RhoU residues 47–204 for stabilization, potentially because it enables folding of the central Rho domain.

To enable investigation into the functional significance of RhoU dimerization, we explored whether RhoU(231–258) could be utilized as a competitive inhibitor. Overexpression of RhoU(231–258)_mut_, which harbors the mutations C255S and C256S and cannot interact with full-length RhoU, had no effect on the co-immunoprecipitation of HA–RhoU with Myc–RhoU. In contrast, overexpression of wild-type RhoU(231–258) significantly impaired the association of the differently tagged full-length RhoU constructs (Fig. 3D).

### Inhibition of self-association attenuates RhoU signaling

**Fig. 4.**
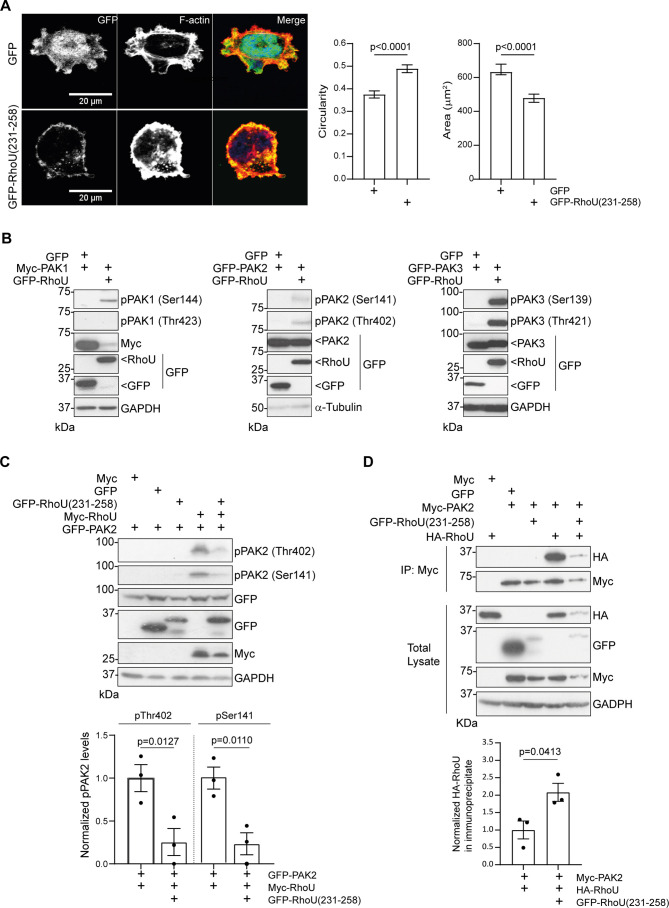
**Inhibition of homo-oligomerization attenuates RhoU signaling.** (A) PC3 cells were transfected with either pEGFP-C1 empty vector or pEGFP-C1-RhoU(231-258) for 16 h and then seeded onto fibronectin-coated glass coverslips for 24 h prior to fixation and staining for F-actin (Alexa Fluor 546 phalloidin, red) and DNA (Hoechst 33342, blue). Images are representative of three independent experiments. Scale bars: 20 µm. Circularity and area were calculated for 120 cells per condition, across three experiments. Data are presented as mean±s.e.m. *P*-values for cell circularity were determined by unpaired two-tailed *t*-test. *P*-values for cell area were determined by Mann–Whitney test. (B) PC3 cells were transfected with pCMV6M-PAK1, pEGFP-C1-PAK2 or pEGFP-C1-PAK3 in combination with pEGFP-C1-RhoU or pEGFP-C1 empty vector and lysates were immunoblotted using anti-GFP and anti-Myc antibodies, an antibody recognizing phosphorylated (p)PAK1(Ser144), pPAK2(Ser141) or pPAK3(Ser139), and an antibody recognizing pPAK1(Thr423), pPAK2(Thr402) or pPAK3(Thr421). GAPDH and α-tubulin were used as loading controls. Blots representative of three repeats. (C) Lysates of COS7 cells transfected with pRK5-myc, pRK5-myc-RhoU, pEGFP-C1, pEGFP-C1-RhoU(231-258) and pEGFP-C1-PAK2 were immunoblotted using anti-GFP, anti-Myc, anti-pPAK2(Thr402) and anti-pPAK2(Ser141) antibodies. GAPDH was using as a loading control. Graph shows mean±s.e.m. values from three independent experiments. *P*-values were determined by one-way ANOVA with Sidak's multiple comparisons post test. (D) Lysates of COS7 cells transfected with pRK5-myc, pRK5-myc-PAK2, HA-RhoU, pEGFP-C1 and pEGFP-C1-RhoU(231-258) were immunoprecipitated (IP) using Myc-TRAP^®^ agarose beads. Samples were immunoblotted using anti-GFP, anti-Myc and anti-HA antibodies. GAPDH was used as a loading control. Graph shows mean±s.e.m. values from three independent experiments. *P*-values were determined by an unpaired two-tailed *t*-test.

Given that RhoU is an atypical GTPase, we considered the possibility that self-association could be required for its full activation. To investigate this, RhoU(231–258) was used as an inhibitor of endogenous RhoU self-association and PC3 cell elongation was used as a read-out of RhoU signaling. PC3 cells expressing GFP–RhoU(231–258) were significantly less elongated and had a smaller spread area than control cells (Fig. 4A), indicating that homo-oligomerization of endogenous RhoU is required for cells to adopt an elongated, migratory phenotype. RhoU has been reported to interact with several proteins, of which the PAKs are the most well characterized (Shutes et al., 2004; Tao et al., 2001). PAKs are serine/threonine kinases that are known to be involved in a variety of cellular responses, including cell morphology and migration. RhoU has been reported to bind to and activate the type I PAK, PAK1 (Saras et al., 2004; Tao et al., 2001), and we found that autophosphorylation of all three type 1 PAKs, PAK1–PAK3, is stimulated by RhoU overexpression in PC3 cells (Fig. 4B). Co-expression with RhoU(231–258) significantly reduced RhoU-stimulated PAK2 autophosphorylation on Thr402 and Ser141 (Fig. 4C), suggesting that oligomerization of full-length RhoU molecules facilitates the activation of PAK2 by RhoU. The interaction of PAK2 with full-length RhoU was slightly increased in cells co-expressing RhoU(231–258), indicating that RhoU self-association is not a pre-requisite for binding to PAK2 (Fig. 4D).

### Concluding remarks

Here, we report for the first time that the atypical Rho GTPase RhoU homodimerizes at the plasma membrane, and that this self-association is required for RhoU-mediated cell elongation and downstream signaling to its target PAK2. The classical Rho GTPase Rac1 and Ras proteins can form multimeric nanoclusters in the plasma membrane (Maxwell et al., 2018; Simanshu et al., 2023), and K-Ras has been reported to homodimerize (Muratcioglu et al., 2015). Our data indicate that RhoU homodimerizes via a distinct mechanism. For example, Rac1 nanoclustering has been shown to be regulated by a key arginine residue within the C-terminal polybasic region, yet mutagenesis of individual charged residues in the conserved RhoU polybasic region had no effect on RhoU self-association (data not shown). Furthermore, the segregation of Rac1 into nanoclusters is influenced by guanine nucleotide-binding status, yet we show that RhoU self-association is mediated by the C-terminal 27 amino acids. Our results therefore demonstrate for the first time that the activity of a Rho family member is stimulated by homodimerization.

## MATERIALS AND METHODS

### Cell culture and treatments

PC3 cells were a kind gift from Magali Williamson (King's College London) and were authenticated by Eurofins-Genomics. Jurkat cells were purchased from the ATCC (Bhavsar et al., 2013). Both cell lines were cultured in Dulbecco's RPMI (Gibco; Thermo Fisher Scientific, 52400-025) supplemented with 10% fetal calf serum (FCS), penicillin (90 U/ml) and streptomycin (90 µg/ml) (Gibco; Thermo Fisher Scientific, 15140-122). HEK293T cells were a gift from Ivan Gout (UCL, London, UK) and were authenticated by Eurofins Genomics. COS7 cells were kindly provided by Kay Mistry (UCL, London, UK). Both cell lines were cultured in Dulbecco's DMEM (Gibco; Thermo Fisher Scientific, 42430-025) supplemented with 10% FCS, penicillin (90 U/ml) and streptomycin (90 µg/ml). Where indicated, cells were treated with 1 µg/ml doxycycline hyclate (Sigma Aldrich, D9891), 10 ng/ml HGF (Peprotech, 100-39), 1 ng/ml CXCL12 (Peprotech, 300-28A) or 100 µM 2-bromopalmitic acid (Focus Biomolecules, 10-3284).

### Cell transfection

HEK293T, COS7 and PC3 cells were transfected with plasmid DNA using Lipofectamine™ 2000 (Invitrogen; Thermo Fisher Scientific, 11668019) according to manufacturer's instructions. Jurkat cells were nucleofected using a Nucleofector II unit (Amaxa Biosystems) together with the Amaxa Cell Line Nucleofector Kit V (Lonza, VVCA-1003). 10^6^ Jurkat cells were suspended in 100 µl Nucleofector Solution and mixed with 2 µg plasmid DNA prior to nucleofection using program X-005. Samples were incubated at room temperature for 10 min, then mixed with 1.5 ml pre-warmed culture medium and incubated at 37°C, 5% CO_2_ for 24 h.

### Generation of doxycycline-inducible stable cell lines

HEK293T cells were transfected at 70% confluency with pMD2.G (Addgene #12259), pCMVΔR8.74 (Addgene #22036) and either empty pINDUCER21 (ORF-EG) (Addgene #46948) or pINDUCER21-Myc-RhoU (see below). The transfection mixture was removed after 16 h and replaced with DMEM plus 10% FBS. After a further 30 h, viral supernatant was harvested and passed through a 0.45 µm pore filter unit. Filtered viral supernatant was mixed with the appropriate cell culture medium at a ratio of 1:2 and applied to either PC3 or DU145 cells at 70% confluency for 24 h. Cells were then expanded, and transduced cells with constitutive GFP expression were sorted by FACS.

### Immunofluorescence and cell shape analysis

PC3 cells containing doxycycline-inducible expression constructs were seeded onto glass coverslips coated with 10 µg/ml fibronectin (Sigma Aldrich, 341365) in 24-well plates at a density of 5000 cells/well and incubated for 4 h at 37°C, 5% CO_2_ before treatment with either 1 µg/ml doxycycline or vehicle control. After 24 h, cells were fixed with 4% paraformaldehyde solution in PBS for 20 min, permeabilized with 0.1% Triton X-100 in PBS for 15 min and blocked in PBS containing 10% FBS and 2% bovine serum albumin (BSA) for 1 h. Cells were then incubated for 1 h with Alexa Fluor 546-conjugated phalloidin (Thermo Fisher Scientific, A22283), fluorescein isothiocyanate (FITC)-labeled mouse α-tubulin antibody (Sigma Aldrich, F2168) and Hoechst 33342 (Thermo Fisher Scientific, 62249). Coverslips were mounted on slides in fluorescence mounting medium (Dako, S3023) and *Z*-stack images with a 1 µm slice interval were generated with a Zeiss LSM Zen 510 confocal microscope using a 40×/1.3 NA objective and Zen software.

PC3 cells transiently transfected with pEGFP-C1 constructs (see below) were seeded onto fibronectin-coated coverslips for 16 h prior to fixation, blocking and permeabilization as above. Cells were stained with Alexa Fluor 546-conjugated phalloidin and Hoechst 33342 and single-plane images of GFP-positive cells were generated as above.

Cell circularity was measured from merged-channel images using the FIJI shape descriptors plugin, where circularity=4π(area/perimeter^2^). A circularity value of 1.0 indicates a perfect circle; as the value approaches 0.0, it indicates an increasingly elongated shape.

### Time-lapse microscopy and random migration analysis

Cells were seeded into 24-well plates coated with 10 µg/ml fibronectin at a density of 5000 cells/well and incubated for 4 h at 37°C, 5% CO_2_ before treatment with either 1 µg/ml doxycycline or vehicle control. After 16 h, cells were imaged every 6 min for 24 h on a Leica DMi8 microscope using a 20× objective at 37°C, 5% CO_2_. Three independent experiments were performed in technical triplicate, with six fields of view (FOVs; 633 μm×633 μm, 1024×1024 pixels) imaged per well. Migration tracks were generated using the MOSES framework, as described previously (Zhou et al., 2019b). Briefly, 5000 superpixel tracks were extracted, tracking forwards from the start of the movie to the last frame. Individual cells in the first frame were segmented by thresholding the non-local means denoised and background subtracted image (background estimated by the white top-hat transform) with morphological postprocessing. The migration track of each unique cell is then given by the most migrating superpixel track covered by its area as previously described (Zhou et al., 2019a). At least 960 cell tracks were analyzed per condition and data were analyzed by Mann–Whitney test.

### Transwell invasion assay

Transwell inserts (Greiner, 665638) in 12-well plates were coated with 300 μg/ml Matrigel (Corning, 354234) diluted in serum-free medium (SFM). Matrigel was set for 2 h at 37°C, 5% CO_2_. 2.5×10^4^ PC3 or DU145 cells were seeded onto each insert in 400 μl SFM supplemented with 0.1% BSA and either 1 µg/ml doxycycline or vehicle control. The basal chamber of each well was filled with 900 µl SFM plus 0.1% BSA supplemented with 10 ng/ml HGF and either 1 µg/ml doxycycline or vehicle control and plates were incubated at 37°C, 5% CO_2_ for 24 h. Material was removed from the apical side of the Transwell filters using a cotton bud prior to submersion in 0.1% Crystal Violet in methanol for 30 min. Filters were washed with distilled water and removed from the Transwell insert using a scalpel before mounting onto glass slides using mounting medium (Dako, S3023). A Leica S9i light microscope was used to image eight FOVs per filter at 50× magnification. Invading cells were quantified by using FIJI to calculate the area covered by cells in each FOV. The mean cell area across all FOVs within each well was then expressed as a fold change over the control condition.

### FLIM and FRET analysis

PC3 cells were transfected for 18 h prior to seeding into 35-mm glass-bottom dishes (Greiner, 627861) coated with 10 μg/ml fibronectin at a density of 1.5×10^5^ cells per dish. After 4 h, adhered cells were fixed for 15 min in 4% paraformaldehyde in PBS. For Jurkat experiments, 10^6^ cells were seeded into each dish 24 h after nucleofection. Cells were seeded in medium supplemented with 1 ng/ml CXCL12 and allowed to adhere for 15 min prior to fixation in 4% paraformaldehyde in PBS for 15 min.

Fluorescence lifetime images were acquired on a Leica TCS SP8 system attached to a Leica DMi8 inverted microscope (Leica Microsystems). Excitation was provided by a white light laser with a repetition rate of 20 MHz and an acousto-optical beam splitter (AOBS) selected an excitation wavelength of 488 nm. Images were acquired using a 63×1.4 NA oil immersion objective. Fluorescence was detected using a hybrid detector operating in photon counting mode over an emission range of 498–535 nm. A notch filter centered on 488 nm minimized any laser scatter into the detector. Time resolved data were acquired through use of a PicoHarp 300 TCSPC module (PicoQuant) controlled through SymPhoTime64 software (PicoQuant). FLIM Images were acquired with 256×256 pixels and 4096 time bins. The total integration time per image was ∼200 s. Fitting of FLIM images was performed with the FLIMfit software tool (version 5.0.3) developed at Imperial College London (Warren et al., 2013). Fitting of the fluorescence images was performed using global analysis across all repeat cells per condition. Data were fitted with a double exponential model on all pixels above an intensity threshold with a 5×5 smoothing kernel applied. All lifetimes are reported as the intensity weighted mean lifetime. Cells with a χ^2^ value >1.5 were excluded.

### DSS crosslinking

COS7 cells were grown in 60-mm dishes and transfected with plasmid DNA at 80% confluency. After 16 h, cells were washed with PBS (Gibco, Thermo Fisher Scientific, 14190094) and lysed in 80 µl conjugation buffer (20 mM HEPES pH 7.2, 1% Triton X-100). Lysates were clarified at 17,000 ***g*** for 10 min at 4°C and supernatant was incubated with either 0.25 mM DSS (Pierce, Thermo Fisher Scientific, A39267) or DMSO vehicle control for 30 min at 4°C with constant rotation. Reactions were quenched by addition of 20 mM Tris-HCl pH 7.5 for 15 min on ice. Samples were mixed with sample buffer (62.5 mM Tris-HCl pH 6.8, 2% SDS, 10% glycerol, 0.2% bromophenol blue, 100 mM DTT) and resolved by SDS-PAGE and immunoblotting.

### Immunoprecipitation

Cells were grown in 100-mm dishes and transfected at 80% confluency. After 16-18 h, cells were then washed in PBS (Gibco, Thermo Fisher Scientific, 14190094) and lysed in 180 µl IP lysis buffer (10 mM Tris-HCl [pH 7.5], 150 mM NaCl, 0.5% NP40) supplemented with protease inhibitor cocktail (Roche, 04693159001) and phosphatase inhibitor cocktail (Roche, 04906837001). Lysates were clarified at 17,000 ***g*** for 10 min at 4°C and supernatants were then mixed with 300 µl IP dilution buffer (10 mM Tris-HCl pH 7.5, 150 mM NaCl) supplemented with protease and phosphatase inhibitor cocktail. Lysates were then incubated with either 25 µl GFP-TRAP^®^ (Chromotek, gtma-20) or Myc-TRAP (Chromoteck, ytma-20) magnetic agarose bead slurry for 1 h at 4°C with constant rotation. Beads were washed three times in IP dilution buffer before protein complexes were eluted in 50 µl 200 mM glycine pH 2.5, for 10 min at 4°C. Total cell lysate and IP samples were mixed with NuPAGE® LDS Sample Buffer (Invitrogen; Thermo Fisher Scientific, NP0008) supplemented with 5% β-mercaptoethanol prior to SDS-PAGE and immunoblotting.

### Immunoblotting

Cells were washed in PBS (Gibco, Thermo Fisher Scientific, 14190094) and lysed in lysis buffer (50 mM Tris-HCl pH 7.5, 150 mM NaCl, 1% Triton X-100) supplemented with protease inhibitor cocktail (Roche, 04693159001) and phosphatase inhibitor cocktail (Roche, 04906837001). Lysates were clarified at 17,000 ***g*** for 10 min at 4°C and supernatants were mixed with NuPAGE^®^ LDS Sample Buffer (Invitrogen; Thermo Fisher Scientific, NP0008) supplemented with 5% β-mercaptoethanol. Samples were boiled at 100°C for 5 min and denatured proteins (30-50 µg) were resolved by electrophoresis on SDS-PAGE gels and transferred onto nitrocellulose membrane (GE Healthcare, 10600020) using an XCell II™ Blot Module (Thermo Fisher, EI9051) according to manufacturer's instructions. Nitrocellulose membranes were blocked with 5% non-fat dried skimmed milk (Marvel) and incubated with primary antibody diluted in Tris-buffered saline (TBS), pH 7.4 (Severn Biotech, 20-7320-011), 5% BSA and 0.1% Tween-20 overnight at 4°C. Membranes were then incubated with a species-appropriate horseradish peroxidase (HRP)-conjugated secondary antibody (Dako) before bands were visualized using an enhanced chemiluminescence detection kit (Cytiva, RPN2209). Primary antibodies against the following proteins were used: c-Myc (Santa Cruz Biotechnology, sc-40, 1:200), GFP (Santa Cruz Biotechnology, sc-9996, 1:200), HA (Proteintech, 51064-2AP, 1:5000), phospho-PAK1 (Thr423)/PAK2 (Thr402) (Cell Signaling Technology, 2601, 1:1000), phospho-PAK1 (Ser199/204)/PAK2 (Ser192/197) (Cell Signaling Technology, 2605, 1:1000), GAPDH (Cell Signaling Technology, 5174, 1:15,000) and α-tubulin (Sigma Aldrich, T5168, 1:10,000). Full images of blots presented in this work can be seen in Fig. S1.

### Expression vectors, cloning and site-directed mutagenesis

pRK5-myc-RhoU vector was a kind gift from Pontus Aspenström (University of Uppsala, Sweden). RhoU cDNA was subcloned from pRK5-myc-RhoU to a pEGFP-C1 vector (Clontech). Briefly, pEGFP-C1 was digested using BamHI and DraI restriction enzymes, whereas pRK5-myc-RhoU was digested using BamHI and PsiI (New England Biolabs). After agarose gel purification, RhoU cDNA was ligated into pEGFP-C1 using T4 DNA ligase (New England Biolabs). RhoU cDNA from pEGFP-RhoU was then subsequently subcloned into a pmCherry-C1 vector using EcoRI restriction enzyme digestion. RhoU(204–258) and RhoU(231–258) coding sequences were amplified by PCR using DNA primers harboring BamHI and XbaI recognition sequences. PCR products and empty pEGFP-C1 were digested with BamHI and XbaI and ligated using T4 DNA ligase. HA–RhoU and GFP–ΔN-RhoU (amino acids 45–258) vectors were kindly provided by Claire Wells (King's College London, UK) (Dart et al., 2015). Point mutations in RhoU cDNA were introduced using a QuikChange II site-directed mutagenesis kit (Agilent, 200523) according to the manufacturer's instructions. Mutated sequences were verified by Sanger sequencing (Eurofins). New vectors can be obtained from our laboratory.

To generate a doxycycline-inducible Myc-RhoU expression construct, the full coding sequence for human RhoU (NM_021205.5) with an N-terminal Myc tag (EQKLISEEDL) was amplified by PCR from pRK5-myc-RhoU and cloned into pENTR™/D-TOPO^®^ according to manufacturer's instructions (Thermo Fisher Scientific, 45-0218). The Myc–RhoU coding sequence was then inserted into pINDUCER21 (ORF-EG) using Gateway™ LR Clonase™ II (Thermo Fisher Scientific, 11791-020).

pcDNA3.1-RhoB was purchased from the cDNA Resource Center. To generate pRK5-myc-RhoB, RhoB cDNA (NM_004040.3) was amplified from pcDNA3.1-RhoB by PCR using DNA primers harboring BamHI and EcoRI recognition sequences. The PCR product and empty pRK5-myc were both digested with BamHI and EcoRI and ligated using T4 DNA ligase. To generate pmCherry-RhoB, RhoB cDNA was amplified by PCR using DNA primers harboring EcoRI and KpnI recognition sequences. The PCR product and empty pmCherry were digested with EcoRI and KpnI and ligated using T4 DNA ligase. pCB6-GFP-RhoB was kindly provided by Ferran Valderrama (St. George's, University of London, UK).

pCMV6M-PAK1 was a kind gift from Jonathan Chernoff (Fox Chase Cancer Center, Philadelphia, PA, USA). pEGFP-C1-PAK2 was kindly provided by Claire Wells (King's College London, UK) and pcDNA3.1-HA-PAK3 by Rick Cerione (Cornell University). To generate pEGFP-C1-PAK3, pcDNA3.1-PAK3 and empty pEGFP-C1 were digested with KpnI and ApaI. After agarose gel purification, PAK3 cDNA (NM_019210.1) was ligated using T4 DNA ligase.

## Supplementary Material



10.1242/joces.261645_sup1 Supplementary information
