# Supplementary figures and images for: RhoU forms homo-oligomers to regulate cellular responses
Source: J Cell Sci. 2024 Jan 30;137(2):jcs261645. doi: 10.1242/jcs.261645 (PMC10917059; doi:10.1242/jcs.261645)

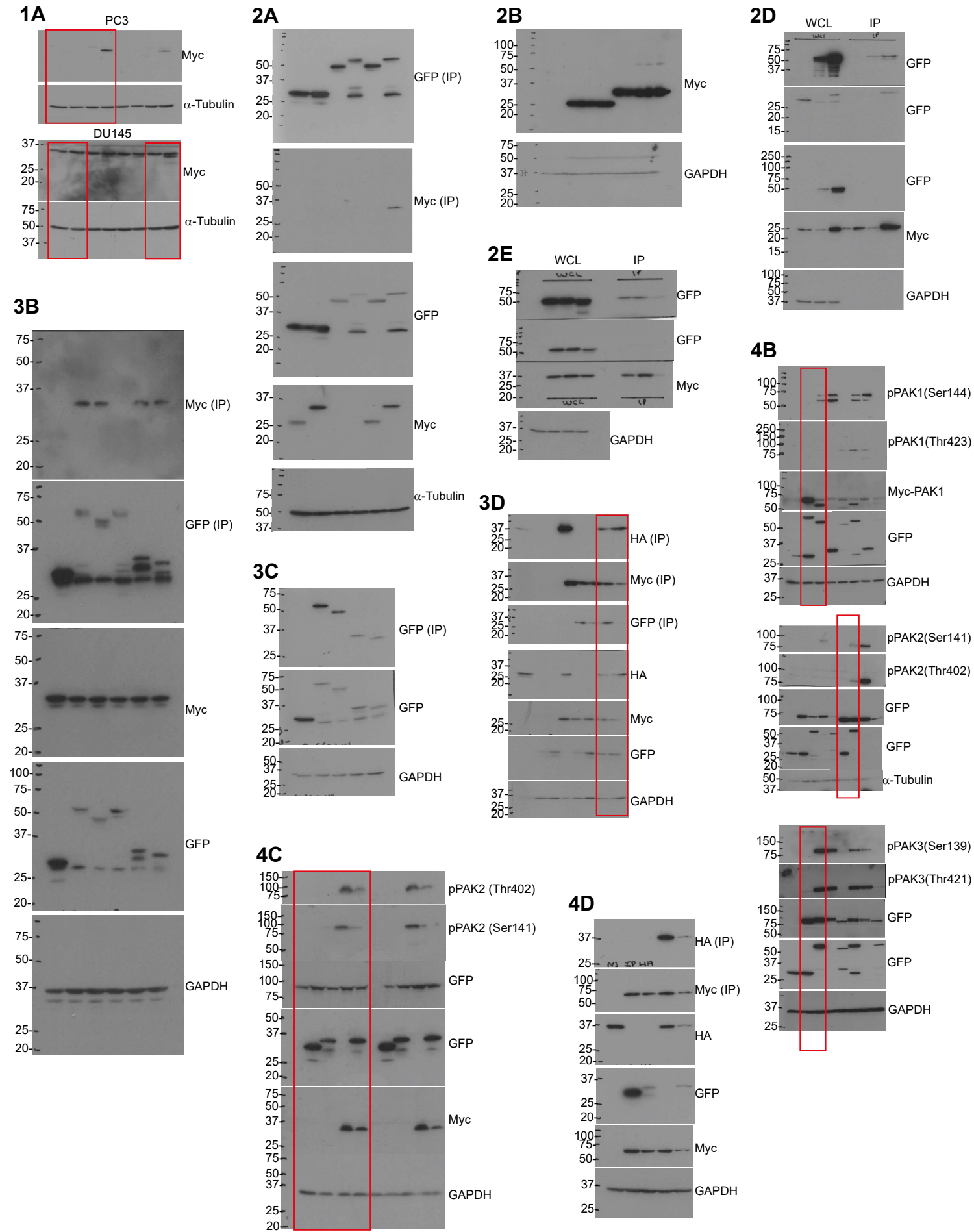

Fig. S1. Blot transparency

Supplement: Supplementary information [file joces-137-261645-s1.pdf]
