# Supplementary material for: RhoU forms homo-oligomers to regulate cellular responses
Source: J Cell Sci. 2024 Jan 30;137(2):jcs261645. doi: 10.1242/jcs.261645 (PMC10917059; doi:10.1242/jcs.261645)
Supplement: Supplementary file 1 [file joces-137-261645_review_history.pdf]

RhoU forms homo-oligomers to regulate cellular responses

Natasha S. Clayton, Richard G. Hodge, Elvira Infante, Dominic Alibhai, Felix Zhou and Anne J. Ridley

DOI: 10.1242/jcs.261645

Editor: Michael Way

Review timeline

Original submission:	13 September 2023
Editorial decision:	12 October 2023
First revision received:	20 December 2023
Accepted:	21 December 2023

Original submission

First decision letter

MS ID#: JOCES/2023/261645

MS TITLE: RhoU forms homo-oligomers to regulate cellular responses

AUTHORS: Natasha S Clayton, Richard G Hodge, Elvira Infante, Dominic Alibhai, Felix Y Zhou, and Anne J Ridley

ARTICLE TYPE: Short Report

We have now reached a decision on the above manuscript.

To see the reviewers' reports and a copy of this decision letter, please go to: https://submit-jcs.biologists.organd click on the 'Manuscripts with Decisions' queue in the Author Area. (Corresponding author only has access to reviews.)

As you will see, the reviewers raise a number of points that prevent me from accepting the paper at this stage. They suggest, however, that a revised version might prove acceptable, if you can address their concerns. I think the points and clarifications that have been raised will strengthen the papers message so you should fully address the reviewers questions. If you think that you can deal satisfactorily with the criticisms on revision, I would be pleased to see a revised manuscript.

Please ensure that you clearly highlight all changes made in the revised manuscript. Please avoid using 'Tracked changes' in Word files as these are lost in PDF conversion.

I should be grateful if you would also provide a point-by-point response detailing how you have dealt with the points raised by the reviewers in the 'Response to Reviewers' box. Please attend to all of the reviewers' comments. If you do not agree with any of their criticisms or suggestions please explain clearly why this is so.

Reviewer 1

Advance summary and potential significance to field

© 2023. Published by The Company of Biologists under the terms of the Creative Commons Attribution License (https://creativecommons.org/licenses/by/4.0/).

This article by Natasha Clayton et al. demonstrate that homo-oligomerization of the atypical Rho GTPase RhoU has in important impact on the signalling capacity of RhoU in prostate cancer cell lines. To my knowledge, this is the first demonstration of oligomerization of a member of the Rho GTPases and this finding might have important impact on our interpretation of past and present literature on signalling by Rho GTPases. This finding is therefore of general interest for all researchers working on Rho GTPase signalling.

Comments for the author

I do not have so much to question in the study. The article is well-written organized with a logical flow, which makes it easy to follow. In my view, the study is worth publishing in JCS after a revision. I have some comments and questions, mostly related to the statistical analysis of the Western blots.

1. Figure 3B: What is the normalization of the RhoU levels supposed to show? As far as I understand, there are no significant differences; the error bars are quite massive even though they represent SEM. In addition, the authors do not say anything about this quantification in the Results section.

2. Figure 3D. This figure is a bit confusing, since at first glance one gets the impression that 2-BP results in an increased interaction between Myc-RhoU and GFP-RhoU. However, the normalization produces the opposite result. I think this is due to the substantial difference in the expression level in the Myc-RhoU+GFP-RhoU control (the middle lane). This is strange, since the same conditions in Figure 3B (second lane from the left). These differences in expression level between different experiments makes the quantification in Figure 3D a bit difficult to believe in. The authors state that the experiment was repeated thrice, was there a consistent difference in RhoU protein levels -/+ 2-BP? If so, what is the reason for this difference?

3. Figure 4D. This is again a counterintuitive result. Just by looking at the HA-RhoU band (the upper band), one gets the impression the RhoU/231-258 polypeptide inhibits the interaction between PAK2 and RhoU. The difference is massive. However, the quantification tells a very different story, again because of a very low expression level of H-RhoU (and GFP-RhoU/231-258). This makes it difficult to trust the result. Was the result the same over the three independent experiments? What is the reason for these rather substantial differences in protein expression?

<u>Reviewer 2</u>

Advance summary and potential significance to field

Clayton et al describe the homo-dimerization of RhoU, member of the Rho GTPase family. They show that RhoU self-associates via its C-terminal part, which needs to be palmitoylated. In cells, overexpression of the Cterminal region has a dominant negative effect, inhibiting RhoU functions. This paper describes a new way of regulation of a Rho GTPase family member.

Majority of the data presented in the manuscript are of good technical quality and the study provides interesting new information about the mechanism by which RhoU is regulated. However, there are several points that should be addressed to strengthen the manuscript.

Comments for the author

Major points

- Figure 1: without dox, PC3-myc-RhoU cells seem to be less circular than control cells. Do the authors have an explanation for that?
It is important to show RhoU overexpression in cells (not only the tag) either by immunoblot or IF.

- Figure 2B: the dose-dependent increase of high-molecular weight myc-RhoU species upon addition of increasing concentrations of DSS is not obvious. Please provide quantification to confirm this statement.

© 2023. Published by The Company of Biologists under the terms of the Creative Commons Attribution License (https://creativecommons.org/licenses/by/4.0/).

Is it possible to see high-molecular weight species for endogenous RhoU in PC3 cells treated with DSS?

- Figures 2C-D: For the FLIM analysis, control experiments with GFP-RhoU and mCherry-alone need to be provided.

- The authors nicely show that the C-terminal region of one monomer is important for the interaction with the other one's.
On Figure 3C, the interaction between Myc-RhoU(231-258) and GFP-RhoU(231-258) appears to be strongly decreased when compared to GFP-RhoU full length. A quantification of this interaction including the 3 independent experiments would help to conclude. Is another region important to stabilize the interaction between the two monomers?
What about the interaction between Myc-RhoU(231-258) and the GFP-RhoU(C255S,C256S)?

- To reinforce the fact that membrane targeting of RhoU is important for its homodimerization, authors should analyse the impact of 2-BP on the interaction detected by FLIM in PC3 cells?

- To confirm that RhoU(231-258) inhibits the homodimerization of RhoU, the authors must perform co-IP between two tagged versions of RhoU (for example, HA-RhoU and GFP-RhoU) in presence or not of myc-RhoU(231-258).

- Even if the RhoU self-association if mediated by its Cter, it would be interesting to analyse the impact of its guanine nucleotide-binding status. Indeed, GTP and GDP-bound forms differ by their 3D structure. Are constitutively active and dominant negative RhoU mutants able to homodimerize?

Minor points

- Figure 2A: the GFP signal is not visible on lanes 1 and 2. Please show a wider view of the GFP immunoblots. As control, it is important to show GFP in the IP fraction (and in the total lysate) to be sure that GFP alone on beads does not immunoprecipitate myc-RhoU.

- Besides Rho GTPases, other small GTPases of the Ras family, such as KRas (PMID 26051715; PMID 29336889), were found to homodimerize. Please discuss this point in the conclusion section.

First revision

Author response to reviewers' comments

Reviewer 1

1. *Figure 3B: What is the normalization of the RhoU levels supposed to show? As far as I understand, there are no significant differences; the error bars are quite massive even though they represent SEM. In addition, the authors do not say anything about this quantification in the Results section.*

The myc-RhoU levels co-immunoprecipitated with various GFP-RhoU mutants was normalized to the levels using wild-type GFP-RhoU in each of the three independent experiments. We changed the blot in Figure 3B for a more representative blot, reflecting that there is no significant difference between levels of myc-RhoU co-immunoprecipitated with any of the truncation mutants. We have added 'ns' to the graph to indicate this. We have added text to the Results section (p. 5, line 94) to state that there was no significant difference for these conditions. We also add that GFP-RhoU(C555S, C556S) mislocalized to the cytosol (p. 5, line 106), consistent with Berzat et al. 2005.

2. *Figure 3D (now Figure 2D in revised manuscript). This figure is a bit confusing, since at first glance one gets the impression that 2-BP results in an increased interaction between Myc-RhoU and GFP- RhoU. However, the normalization produces the opposite result. I think this is due to*

© 2023. Published by The Company of Biologists under the terms of the Creative Commons Attribution License (https://creativecommons.org/licenses/by/4.0/).

the substantial difference in the expression level in the Myc-RhoU+GFP-RhoU control (the middle lane). This is strange, since the same conditions in Figure 3B (second lane from the left). These differences in expression level between different experiments makes the quantification in Figure 3D a bit difficult to believe in. The authors state that the experiment was repeated thrice, was there a consistent difference in RhoU protein levels -/+ 2-BP? If so, what is the reason for this difference?

We have repeated the experiment with 2-BP again, replaced the blot with a new more representative blot, and revised the quantitation to incorporate the results from this repeat. We consistently observe an increase in RhoU protein levels following 2-BP treatment. We have added to the text that 2-BP increases the expression levels of myc-RhoU and GFP-RhoU, which could suggest that cytoplasmic RhoU is more stable (p. 5).

3. Figure 4D. This is again a counterintuitive result. Just by looking at the HA-RhoU band (the upper band), one gets the impression the RhoU/231-258 polypeptide inhibits the interaction between PAK2 and RhoU. The difference is massive. However, the quantification tells a very different story, again because of a very low expression level of H-RhoU (and GFPRhoU/231-258). This makes it difficult to trust the result. Was the result the same over the three independent experiments? What is the reason for these rather substantial differences in protein expression?
Co-expression of Myc-PAK2, GFP-RhoU(231-258) and HA-RhoU results in much lower expression of Myc-PAK2 and HA-RhoU in all three repeats of the experiment. This is why the densitometry analysis is essential. We believe this is to do with the epitope tags because co-expression of GFP-RhoU(231- 258), Myc-RhoU and GFP-PAK2 does not have the same effect on GFP-PAK2 or myc-RhoU expression levels in Fig 4C.

Reviewer 2

1. Figure 1: without dox, PC3-myc-RhoU cells seem to be less circular than control cells. Do the authors have an explanation for that? It is important to show RhoU overexpression in cells (not only the tag) either by immunoblot or IF.
The control and myc-RhoU cells are separate PC3 populations with their own intrinsic variability (as is frequently observed with different populations derived from the same cell line, noting the many distinct populations of HeLa cells across laboratories). The two populations were never intended to be compared with each other.
[Editorial note: in the above response, the author intended to refer to PC3 cells, not HeLa cells]
We have tested two different commercial RhoU antibodies extensively, and unfortunately neither of them recognizes endogenous RhoU, so it has not been possible to compare the level of RhoU overexpression in Myc-RhoU-expressing cells.

2. Figure 2B: the dose-dependent increase of high-molecular weight myc-RhoU species upon addition of increasing concentrations of DSS is not obvious. Please provide quantification to confirm this statement. Is it possible to see high molecular weight species for endogenous RhoU in PC3 cells treated with DSS?
We have added quantification of the DSS crosslinking data with p values to Figure 2B and changed the blot to a more representative one with increasing levels of dimeric myc-RhoU in response to DSS concentration.
As mentioned in our answer to point 1 above, we tested two commercial antibodies to RhoU extensively under different conditions. One did not detect any bands on western blots; the other produced multiple non-specific bands, including at the predicted molecular weight of RhoU dimers.

3. Figures 2C-D (now Figure 2C): For the FLIM analysis, control experiments with GFP-RhoU and mCherry-alone need to be provided.
We included this control for RhoU in Jurkat cells (Figure 2C), but free mCherry was expressed at much higher levels than mCherry-RhoU (Jurkat cells and PC3 cells, see figure below for PC3 cells) or mCherry-RhoB (PC3 cells), which appeared to produce some artificial FRET. We argue that the mCherry-RhoB is a much better negative control for mCherry-RhoU than mCherry alone, because it is a Rho GTPase that can also be palmitoylated, like RhoU, and localize to membranes, but does not dimerize/oligomerize (p. 4, lines 65-67).

© 2023. Published by The Company of Biologists under the terms of the Creative Commons Attribution License (https://creativecommons.org/licenses/by/4.0/).

[NOTE: We have removed unpublished data that had been provided for the referees in confidence]

4. Figure 3C. The authors nicely show that the C-terminal region of one monomer is important for the interaction with the other one's. On Figure 3C, the interaction between Myc-RhoU(231-258) and GFP- RhoU(231-258) appears to be strongly decreased when compared to GFP-RhoU full length. A quantification of this interaction including the 3 independent experiments would help to conclude. Is another region important to stabilize the interaction between the two monomers? What about the interaction between Myc-RhoU(231-258) and the GFP-RhoU(C255S,C256S)?
We repeated this experiment twice to obtain better blots for quantification, which has now been added to Figure 3C with p values. This demonstrates that indeed the interaction between myc-RhoU(231-258) and GFP-RhoU(231-258) is indeed lower than with full-length GFP-RhoU. We therefore suggest that while the C-terminal region mediates dimerization, the region between RhoU(47-204) could stabilize this interaction by correct folding of the Rho domain (p. 6, lines 115-118).

5. To reinforce the fact that membrane targeting of RhoU is important for its homodimerization, authors should analyse the impact of 2-BP on the interaction detected by FLIM in PC3 cells?
Given that 2-BP treatment increases the expression of GFP-RhoU (see revised Figure 2D and answer to reviewer 1, point 2) and is therefore likely to increase mCherry-RhoU expression. After discussions with our co-author with extensive FLIM expertise (Dominic Alibhi), we were concerned that this experiment would give 'artificial FRET' in the cytoplasm due to the higher expression levels.
We have other data reinforcing the importance of membrane localization for RhoU homodimerization (e.g. co-IPs with RhoU(C255S,C256S mutant; Figure 2B).

6. To confirm that RhoU(231-258) inhibits the homodimerization of RhoU, the authors must perform co-IP between two tagged versions of RhoU (for example, HA-RhoU and GFP-RhoU) in presence or not of myc-RhoU(231-258.)
We carried out this experiment using the double cysteine (C555S, C556S) mutant (of GFP-RhoU(231-258) as the negative control. The new data are included in Figure 3D and described in the results (p. 6, lines 119-124).

7. Even if the RhoU self-association if mediated by its Cter, it would be interesting to analyse the impact of its guanine nucleotide-binding status. Indeed, GTP and GDP-bound forms differ by their 3D structure. Are constitutively active and dominant negative RhoU mutants able to homodimerize? Thank you for suggesting this experiment. We used the RhoU(Q107L) constitutively active mutant, which showed no difference in homodimerization. These new data are added to Figure 2E and described in the results (p. 5, lines 97-99). We did not test the putative 'dominant negative' mutant RhoU(T63N) because it has not been proven to function as a dominant negative protein. Moreover, the different biochemical properties of RhoU (high spontaneous nucleotide exchange; Shutes et al., Curr Biol 2004) compared to classical Rho GTPases (e.g. Rac1, Cdc42, RhoA) means that there is no evidence that it requires a GEF to activate it (which other dominant negative small GTPases bind to and titrate out).

8. Figure 2A: the GFP signal is not visible on lanes 1 and 2. Please show a wider view of the GFP immunoblots. As control, it is important to show GFP in the IP fraction (and in the total lysate) to be sure that GFP alone on beads does not immunoprecipitate myc-RhoU.
We have edited Figure 2A to show the GFP alone, which does not immunoprecipitate GFP-RhoU. Note that we observe some cleavage of the GFP tag from RhoU, since some GFP is visible at a similar molecular weight to GFP alone. We and others have observed this with other GFP-tagged proteins and is not unique to GFP-RhoU.

9. Besides Rho GTPases, other small GTPases of the Ras family, such as KRas (PMID 26051715; PMID 29336889), were found to homodimerize. Please discuss this point in the conclusion section.

We have added a comment to state that Ras proteins have been reported to homodimerize on p. 6,

© 2023. Published by The Company of Biologists under the terms of the Creative Commons Attribution License (https://creativecommons.org/licenses/by/4.0/).

lines 149-150 (although we note this is controversial; PMID: 36990093).

Second decision letter

MS ID#: JOCES/2023/261645

MS TITLE: RhoU forms homo-oligomers to regulate cellular responses

AUTHORS: Natasha S Clayton, Richard G Hodge, Elvira Infante, Dominic Alibhai, Felix Y Zhou, and Anne J Ridley

ARTICLE TYPE: Short Report

I am happy to tell you that your manuscript has been accepted for publication in Journal of Cell Science, pending standard ethics checks.

© 2023. Published by The Company of Biologists under the terms of the Creative Commons Attribution License (https://creativecommons.org/licenses/by/4.0/).
